# Secondary Household Transmission of SARS, Singapore

**DOI:** 10.3201/eid1002.030676

**Published:** 2004-02

**Authors:** Denise Li-Meng Goh, Bee Wah Lee, Kee Seng Chia, Bee Hoon Heng, Mark Chen, Stefan Ma, Chorh Chuan Tan

**Affiliations:** * National University of Singapore, Singapore; † National Healthcare Group, Singapore; ‡ Tan Tock Seng Hospital, Singapore; § Ministry of Health, Singapore

**Keywords:** severe acute respiratory syndrome, coronavirus, secondary, household, transmission, attack rate

## Abstract

Secondary household transmission of severe acute respiratory syndrome (SARS) was studied in 114 households involving 417 contacts. The attack rate was low (6.2%). Occupation of the index case was the factor that most influenced household transmission (adjusted hazard ratio for healthcare workers 0.157; 95% confidence interval 0.042 to 0.588).

Severe acute respiratory syndrome (SARS) is an emerging infectious disease caused by the SARS-associated coronavirus (SARS-CoV) (*1*). Attack rates are >50% in hospitals (*2*). A similar trend was seen in Singapore, with SARS spreading to five hospitals and two Specialty Centres within 8 weeks (*3*). This rapid rate of transmission caused a national health alert and resulted in large amounts of manpower and resources being deployed.

On the other hand, transmission within the household was less efficient. We, therefore, examined the attack rate and the factors influencing secondary transmission of SARS in Singapore households. Data on probable SARS cases were collected by Singapore’s Ministry of Health Epidemiology Unit, Singapore. The case definition of probable SARS was in accordance with the World Health Organization (WHO) (*4*).

Probable SARS cases that were also a household index were identified by using the definition that follow. A household was defined as a residential place with a unique address. A household index was a person with probable SARS and the first person to introduce SARS into the household. A household contact was defined as a person living in the same household as the household index.

Demographic and clinical data were collected. For the household index, the following information was collected: age, sex, if the household index was a healthcare worker (defined as a person who works in a healthcare setting), number of days spent at home after onset of symptoms, and number of contacts in household. For household contacts, the following information was collected: age, sex, if the contact was a healthcare worker, and if the contact was a family member. The week of the SARS outbreak in Singapore was also evaluated to see if there was a time trend in the risk for transmission.

All household contacts were followed prospectively for (*1*) clinical symptoms until 20 days after the last contact with the household index, and (*2*) evidence of positive PCR (polymerase chain reaction) or serologic test for SARS-CoV (according to criteria set by WHO). Secondary household transmission was said to have occurred if the household contact fulfilled the case definition of probable SARS (*4*).

Households were excluded if the household index lived alone, the household index did not spend time at home after onset of symptoms, if the period of household exposure to the index was not clearly defined (e.g., not isolated promptly upon hospital admission), or more than one index lived in the household (shown through contact tracing or onset <2 days after SARS developed in the first person in the household).

Statistical tests (Mann-Whitney, chi-square and Fisher exact test) were used to test for associations when appropriate. The Cox Regression model was used to evaluate the influence of demographic and clinical factors on secondary household transmission. All analyses were performed with SPSS version 11.5.

There were 205 probable SARS cases in Singapore during the period between February 24 and April 29, 2003. These 205 probable SARS cases resided in 163 households. A total of 114 households fulfilled the inclusion and exclusion criteria. Forty-nine households were excluded (12 because the index lived alone, 20 because the household index did not spend time at home after onset of symptoms, 10 because the period of household exposure to the index was not clearly defined, 7 because more than one index patient was in that household). Seventy-two of the 114 household indexes (63.2%) were healthcare workers. Ten were doctors, 37 were nurses, 4 were nursing students, and 21 were paramedical staff.

From these 114 households, 417 household contacts were identified and followed prospectively. Secondary transmission occurred in only 14 households (12.3%), giving rise to 26 household cases of probable SARS. Household transmissions occurred within 2–11 days (mean 5.3 ± 2.6 days) after the onset of symptoms in the index cases. Symptoms developed in eight contacts (30%) while on home quarantine orders. The remaining 18 were not given home quarantine orders because they were either already in hospital with SARS or were not identified by contact tracing. The mean length of stay at home after onset of symptoms was not statistically different between the home-quarantined group and the group not quarantined at home (p = 0.09).

The secondary household attack rate was thus low (6.2% [95% confidence interval 3.9% to 8.6%]) and concurs with that reported by Beijing, China (*5*). In that study, the attack rate was 4.6% in persons who had contact with a probable SARS case-patient during the symptomatic period and lived in the same residence (which included some persons who visited or cared for a SARS patient). These findings are in contrast to the high attack rate seen in the healthcare setting (*6*). One possible explanation for this difference is the phase of the illness. SARS case-patients in the household tend to be in the early phase of illness whereas SARS case-patients in the healthcare settings tend to be in the later phase. In addition, coexisting conditions and invasive procedures done within the hospital setting may also influence risk of transmitting disease (*7*).

The low rate of household transmission suggests that the magnitude of a household outbreak would be less than a hospital-based one, which could help allay public fear and panic, a societal concern evident in the recent outbreak (*2*,*7*). This knowledge will also enable public health officers to develop a more sensitive and responsive surveillance system. As the expected attack rate is known, healthcare professionals can be prepared early if the observed attack rate in the households is higher than predicted, allowing rational rather than empirical implementation of public health measures and justify rapid and aggressive investigative and containment measures needed to prevent a large outbreak. These considerations are particularly important for countries with limited healthcare and fiscal resources. In Singapore, we learned the usefulness of educating persons on the need and means of doing daily temperature monitoring, to have a centralized temperature recording database for hospital staff and patients so that a cluster of fevers could be spotted early, to evaluate symptomatic hospital staff in designated hospital clinics, and to trace contacts by using many resources including the police and army. The authorities in Hong Kong did not have the benefit of this information as little was known then about SARS. Perhaps in the future, such knowledge will help prevent another situation similar to that seen in Amoy Gardens, Hong Kong Special Administrative Region (*8*).

Factors influencing household transmission were also studied in the Singapore cohort. Univariate analysis (Table 1) showed that household index cases were less likely to transmit SARS to their household contacts if they were younger or were healthcare workers. Contacts were more likely to develop SARS if they were not healthcare workers or family members. The Cox regression model (Figure and Table 2) verified two of these four factors, index occupation and age.

**Table 1:** Characteristics of household contacts and index cases^a^

Risk Factor	Household contacts with SARS (n = 26) (mean + 1 SD)	Household contacts without SARS (n = 391) (mean + 1 SD)	p value
Household contact			
Age (y)	35.3 ± 19.8	30.3 ± 17.4	0.17
Sex (female)	14 (53.8%)	225 (57.5%)	0.71
Healthcare worker	1 (3.8%)	84 (21.5%)	0.04
Family member	24 (92.3%)	269 (68.8%)	0.01
Index case			
Age (y)	53.5 ± 16.2	35.4 ± 13.6	<0.001
Sex (female)	20 (76.9%)	290 (74.2%)	0.76
Healthcare worker	4 (15.4%)	273 (69.8%)	<0.001
Days index spent at home after onset of symptoms	5.3 ± 2.5	4.8 ± 2.5	0.43
No. of persons in household	5.0 ± 3.0	4.8 ± 2.4	0.79

^a^Using Univariate analysis, SARS, severe acute respiratory syndrome.

**Figure:**
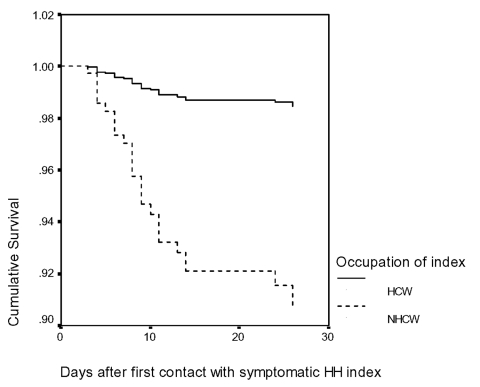
Survival analysis for secondary household transmission (Cox regression model). Household contacts were more likely to get SARS if the index was older or a nonhealthcare worker. Hazard ratios of risk factors analyzed are tabulated in Table 2. The -2log likelihood for this analysis was 253.77. HH, household; HCW, healthcare worker; NHCW, nonhealthcare worker.

**Table 2:** Hazard ratios of risk factors analyzed^a^

Independent risk factor	Hazard ratio (95% CI)	p value
Household contact		
Age (yrs)	1.013 (0.992 to 1.034)	0.222
Sex (female)	1.232 (0.542 to 2.796)	0.619
Healthcare worker	1.692 (0.137 to 20.926)	0.682
Family member	1.936 (0.372 to 10.076)	0.432
Household index		
Age (y)	1.055 (1.015 to 1.097)	0.007
Sex (female)	1.274 (0.451 to 3.595)	0.648
Healthcare worker	0.157 (0.042 to 0.588)	0.006
Days index spent at home after onset of symptoms	0.942 (0.794 to 1.117)	0.493
No. of persons in household	1.060 (0.899 to 1.249)	0.490
Week of outbreak	1.019 (0.733 to 1.417)	0.911

^a^ Using Cox Regression Model; CI, confidence interval.

The most consistent and important factor influencing household transmission was whether or not the index case was a healthcare worker (adjusted hazard ratio 0.157; 95% CI 0.042 to 0.588). This was independent of length of exposure or demographics. The reason for this finding was not evident from the data available. A difference in social behavior between healthcare worker and nonhealthcare worker is a possible explanation for this disparity in risks of household transmission. For example, healthcare workers may be more acutely aware of the risk of acquiring and transmitting SARS and may alter hygiene practices at home. In addition, better health and disease prevention knowledge may influence the efficacy of such practices. Qualitative differences in social behavior between healthcare worker and nonhealthcare worker should be investigated, as this knowledge may be useful in containing future SARS outbreaks.

The risk for household transmission was also lower if the index case was younger. This finding may correlate with milder disease seen in younger persons and lower infectivity. The week of the outbreak did not significantly influence the model, indicating the lack of a time trend in household transmission.

In conclusion, this study is the first to characterize secondary household transmission of SARS. We have shown that the attack rate is low and the most significant factor influencing household transmission was the occupation of the index case. The results of this study challenge some of the current concepts about SARS. Given that the study numbers are not large, a multicenter analysis of the past SARS cases would be helpful in verifying these findings.

## References

[1] Ksiazek TG, Erdman D, Goldsmith CS, Zaki SR, Peret T, Emery S, A novel coronavirus associated with severe acute respiratory syndrome. N Engl J Med. 2003;348:1953. 10.1056/NEJMoa030781 12690092

[2] World Health Organization. Outbreak news—severe acute respiratory syndrome (SARS). Wkly Epidemiol Rec. 2003;78:81.12701272

[3] Hsu L-Y, Lee C-C, Green JA, Ang B, Paton NI, Lee L, Severe acute respiratory syndrome (SARS) in Singapore: clinical features of index patient and initial contacts. Emerg Infect Dis. 2003;9:713–7. 12781012 10.3201/eid0906.030264 PMC3000162

[4] Lingappa JR, McDonald C, Parashar U, Simone P, Anderson L. SARS emergence from uncertainty. Emerg Infect Dis. 2004;▪▪▪:10.

[5] Efficiency of quarantine during an epidemic of severe acute respiratory syndrome—Beijing, China, 2003. MMWR Morb Mortal Wkly Rep. 2003;52:1037. 14586295

[6] World Health Organization. Outbreak news—severe acute respiratory syndrome (SARS). Wkly Epidemiol Rec. 2003;78:81.12701272

[7] Lee N, Hui D, Wu A, Chan P, Cameron P, Joynt GM, A major outbreak of severe acute respiratory syndrome in Hong Kong. N Engl J Med. 2003;348:1986. 10.1056/NEJMoa030685 12682352

[8] H.K.S.A.R.C. Department of Health. Outbreak of Severe Acute Respiratory Syndrome (SARS) at Amoy Gardens, Kowloon Bay, Hong Kong. Main Findings of the Investigation; 2003.

